# Supplementary material for: Rapid Bacterial Recognition over a Wide pH Range by Boronic Acid-Based Ditopic Dendrimer Probes for Gram-Positive Bacteria
Source: Molecules. 2021 Dec 31;27(1):256. doi: 10.3390/molecules27010256 (PMC8746651; doi:10.3390/molecules27010256)
Supplement: Supplementary file 1 [file molecules-27-00256-s001.zip › molecules-1525796-supplementary.pdf]

Rapid Bacterial Recognition over a Wide pH Range by Boronic Acid-Based Ditopic Dendrimer Probes for Gram-Positive Bacteria

Ayame Mikagi,[1] Koichi Manita,[1] Asuka Yoyasu,[1] Yuji Tsuchido,[1,2]

Nobuyuki Kanzawa,[1] Takeshi Hashimoto[1] and Takashi Hayashita[1]*

1. Department of Materials and Life Sciences, Faculty of Science and Technology, Sophia University, 7-1 Kioi-cho, Chiyoda-ku, Tokyo 102-8554, Japan

2. Department of Life Science and Medical Bioscience, School of Advanced Science and Engineering, Waseda University (TWIns), 2-2 Wakamatsu-cho, Shinjuku-ku, Tokyo 162-8480, Japan

*Corresponding author. E-mail address: ta-hayas@sophia.ac.jp (T. Hayashita).

Contents

S1-S8: Analytical Data

S9-S18: ^{1}H NMR and MS Spectra

A) *S. aureus*

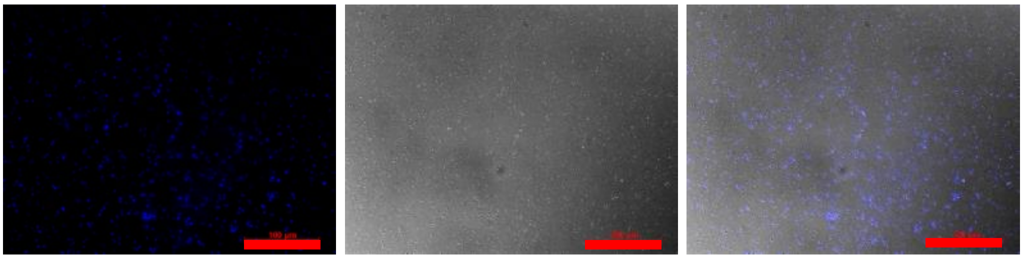

B) *E. coli*

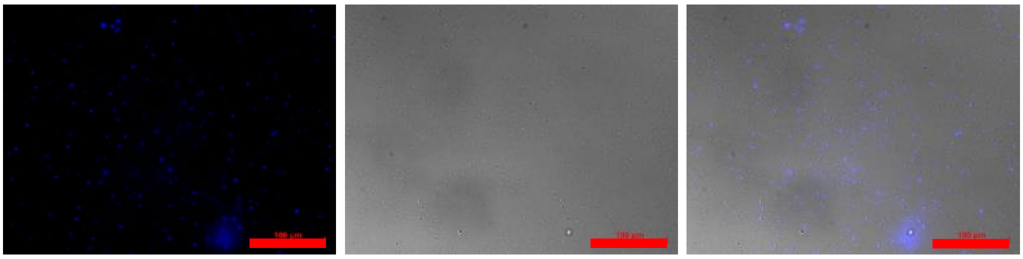

C) *S. aureus* + Cu^{2+} ion

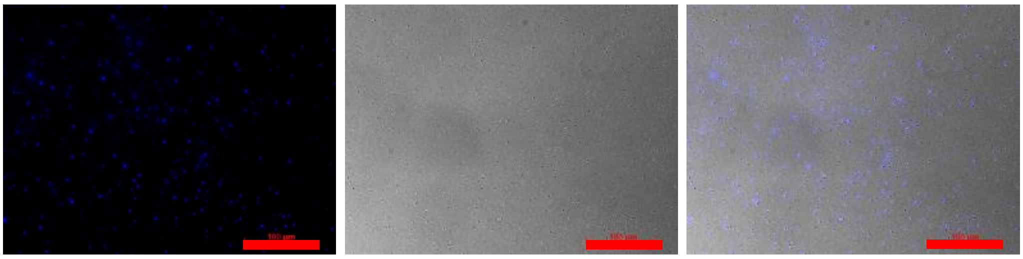

D) *E. coli* + Cu^{2+} ion

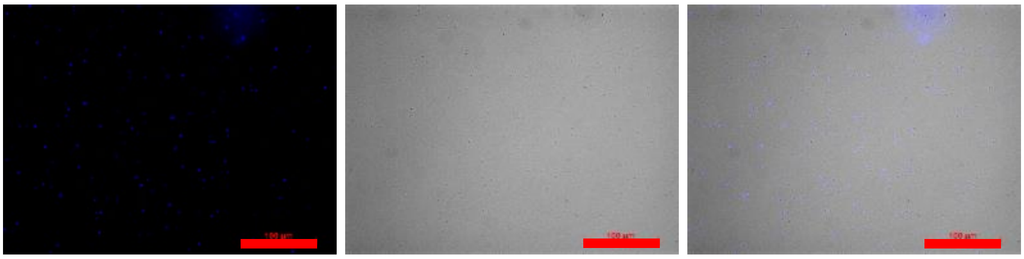

E) *S. aureus* + dpa-PAMAM

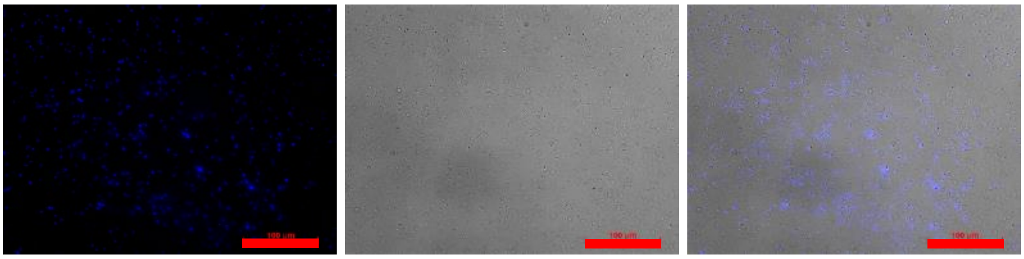

F) *E. coli* + dpa-PAMAM

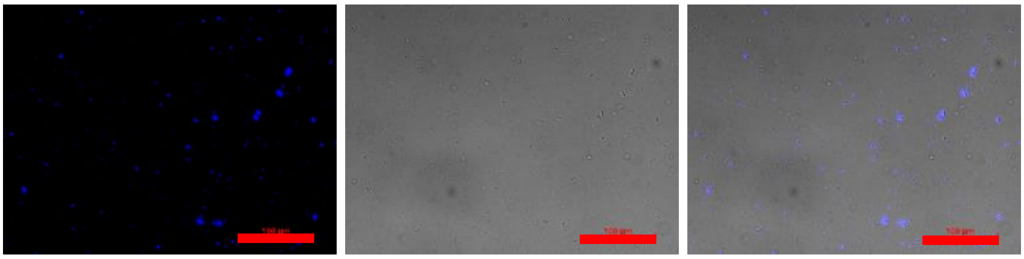

Figure S1. Fluorescent microscopy images of control samples at pH 7.4 adjusted with HEPES buffer. [bacteria] = 2.3 × 10^8 CFU·mL^{-1}, [HEPES] = 5.0 mM, the figures (from left to right) demonstrated DAPI, DIC, and merged images. Scale bar was set to 100 μm. C,D) [$Cu(NO_3)_2$] = 13.2 μM, E,F) [probe] = 3.3 μM. All images (A-F) did not show aggregation.

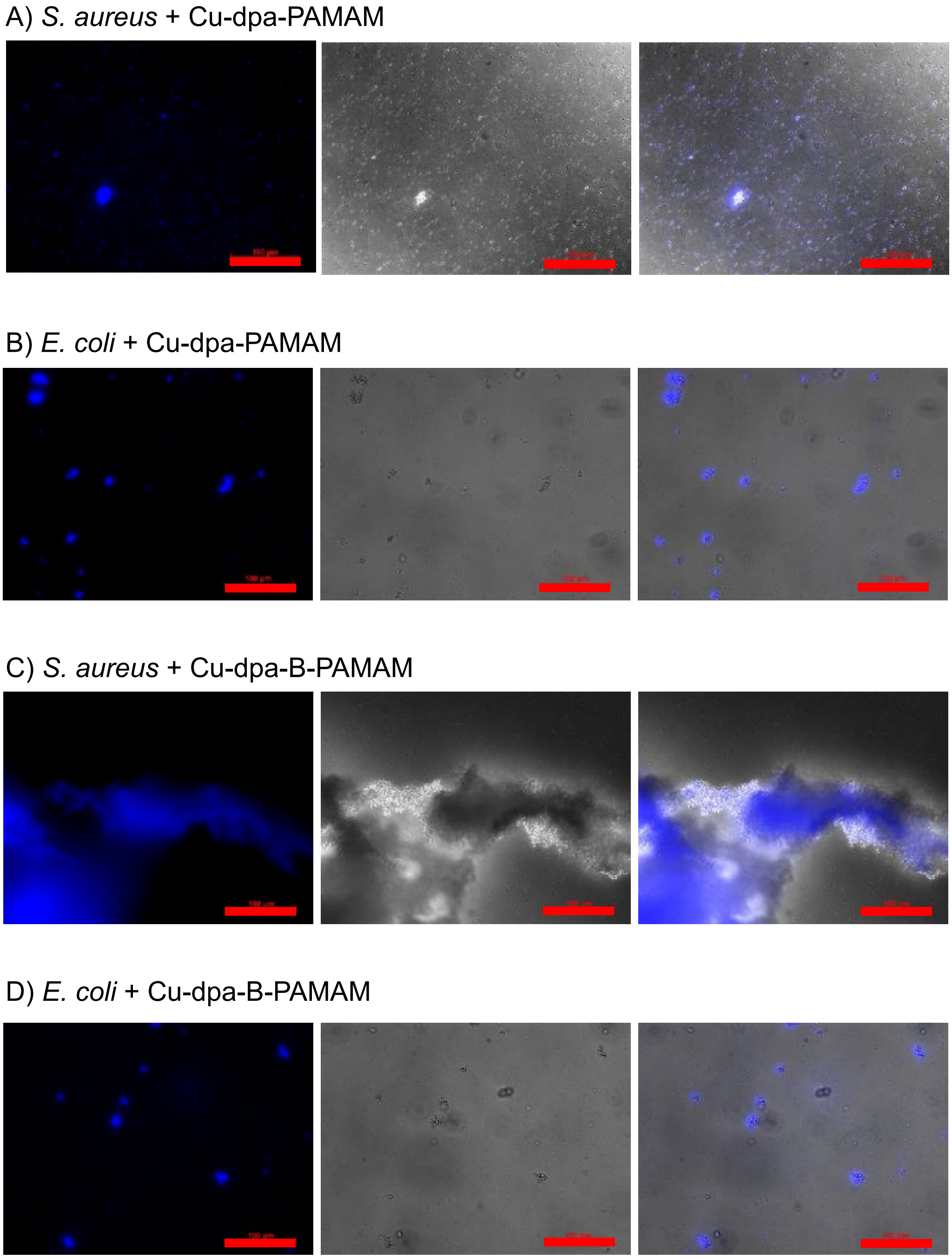

Figure S2. Fluorescent microscopy images of Cu-dpa probes at pH 7.4 adjusted with HEPES buffer. [probe] = 3.3 μM, [Cu^{2+}] = 13.2 μM, [bacteria] = 2.3 × 10^{8} CFU·mL^{-1}, [HEPES] = 5.0 mM, the figures (from left to right) demonstrated DAPI, DIC, and merged images. Scale bar was set to 100 μm. The images (C) gave especially large aggregates.

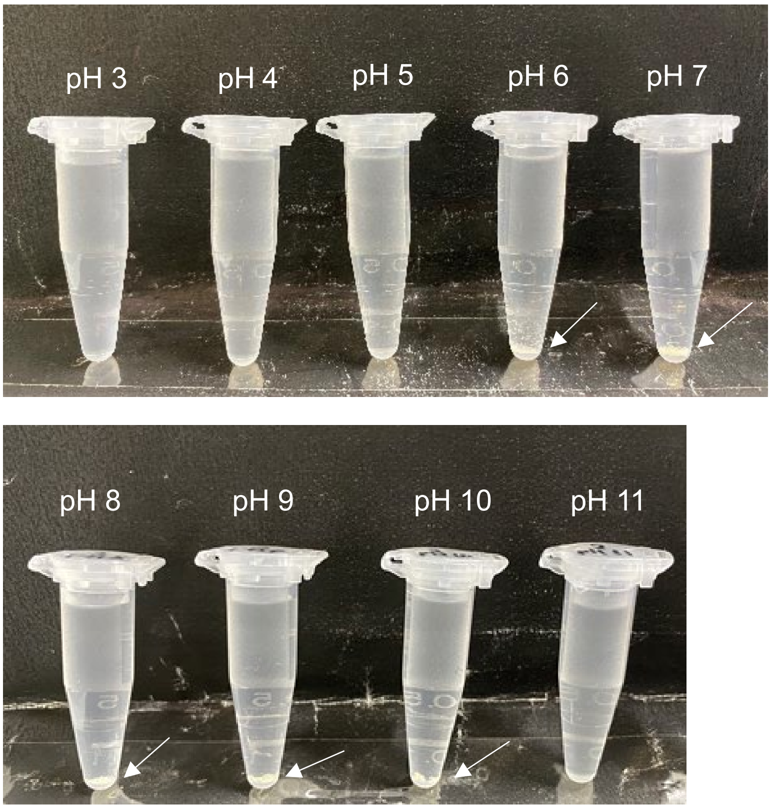

Figure S3. The images of aggregation between Cu-dpa-B-PAMAM and *S. aureus*. [Cu-dpa-B-PAMAM] = 3.3 μM, [Cu^{2+}] = 13.2 μM, [*S. aureus*] = 2.3 × 10^8 CFU·mL^{-1}, [HEPES or CAPS] = 5.0 mM. The results of turbidity measurement are demonstrated in Figure 5A.

Table S1. Investigation of detection limit by turbidity measurements at pH 7.4 adjusted by HEPES buffer (n = 5). [HEPES] = 5.0 mM, [Cu-dpa-B-PAMAM] = 3.3 μM, [Cu^{2+}] = 13.2 μM, [bacteria] = 2.3 × 10^5–2.3 × 10^8 CFU·mL^{-1}. Paired t-test was used to compare OD_{600} (before) and OD_{600} in each concentration. Differences were calculated with two-sided test with an alpha level of 0.05. Asterisk was considered a significant difference (p < 0.05). It must be noted that the conditions with a high concentration of *S. aureus* could be easily discriminated by confirming aggregation that existed as viable precipitation.

Cu-dpa-B-PAMAM with *S. aureus*

	2.3 × 10^5 CFU·mL^{-1}		2.3 × 10^6 CFU·mL^{-1}		2.3 × 10^7 CFU·mL^{-1}		2.3 × 10^8 CFU·mL^{-1}	
	OD_{600} (before)	OD_{600}	OD_{600} (before)	OD_{600}	OD_{600} (before)	OD_{600}	OD_{600} (before)	OD_{600}
1	0.0027	0.0021	0.0078	0.0089	0.0645	0.0798	0.5331	0.0822
2	0.0030	0.0026	0.0074	0.0096	0.0622	0.0787	0.5555	0.0917
3	0.0029	0.0021	0.0078	0.0106	0.0620	0.0782	0.5708	0.0812
4	0.0051	0.0041	0.0080	0.0088	0.0634	0.0791	0.5677	0.0796
5	0.0035	0.0040	0.0080	0.0112	0.0632	0.0771	0.5590	0.0924
Average	0.0034	0.0030	0.0078	0.0098	0.0631	0.0786	0.5572	0.0854
SD	0.0009	0.0009	0.0002	0.0009	0.0009	0.0009	0.0133	0.0055
p-value	-	0.152	-	0.012*	-	4.4×10^{-6}*	-	3.7×10^{-7}*

Cu-dpa-B-PAMAM with *E. coli*

	2.3 × 10^5 CFU·mL^{-1}		2.3 × 10^6 CFU·mL^{-1}		2.3 × 10^7 CFU·mL^{-1}		2.3 × 10^8 CFU·mL^{-1}	
	OD_{600} (before)	OD_{600}	OD_{600} (before)	OD_{600}	OD_{600} (before)	OD_{600}	OD_{600} (before)	OD_{600}
1	0.0027	0.0030	0.0049	0.0056	0.0275	0.0412	0.2449	0.2815
2	0.0030	0.0027	0.0048	0.0043	0.0264	0.0418	0.2405	0.2915
3	0.0025	0.0029	0.0044	0.0054	0.0274	0.0407	0.2519	0.2707
4	0.0028	0.0026	0.0048	0.0041	0.0276	0.0404	0.2481	0.2734
5	0.0029	0.0030	0.0050	0.0042	0.0276	0.0400	0.2403	0.2829
Average	0.0028	0.0028	0.0048	0.0047	0.0273	0.0408	0.0045	0.2800
SD	0.0002	0.0002	0.0002	0.0006	0.0005	0.0006	0.0349	0.0074
p-value	-	0.683	-	0.881	-	1.3×10^{-5}*	-	0.004*

A) *S. aureus* (2.3 × 10^7 CFU·mL^{-1}) + Cu-dpa-B-PAMAM

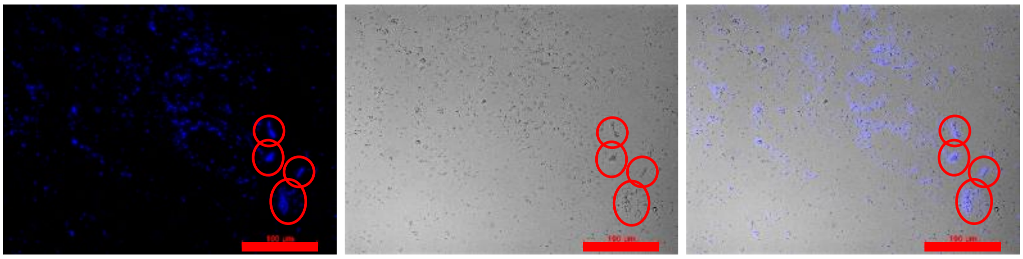

B) *S. aureus* (2.3 × 10^7 CFU·mL^{-1})

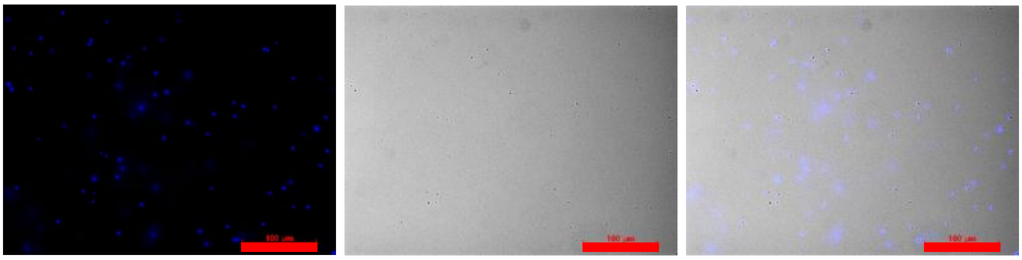

C) *S. aureus* (2.3 × 10^5 CFU·mL^{-1}) + Cu-dpa-B-PAMAM

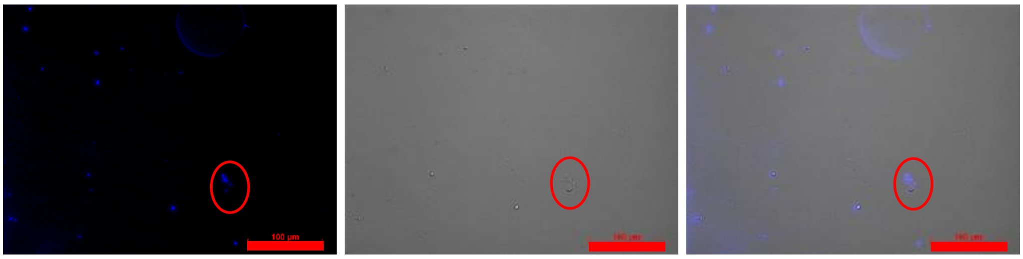

D) *S. aureus* (2.3 × 10^5 CFU·mL^{-1})

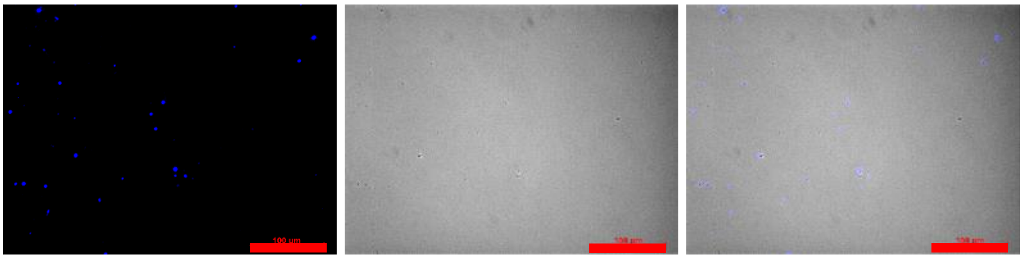

Figure S4. Fluorescent microscopy images in low *S. aureus* concentrations at pH 7.4 adjusted with HEPES buffer. [HEPES] = 5.0 mM, the figures (from left to right) demonstrated DAPI, DIC, and merged images. Scale bar was set to 100 μm. A,B) [*S. aureus*] = 2.3 × 10^7 CFU·mL^{-1}, [Cu-dpa-B-PAMAM] = 3.3 μM or none, [Cu^{2+}] = 13.2 μM or none. C,D) [*S. aureus*] = 2.3 × 10^5 CFU·mL^{-1}, [Cu-dpa-B-PAMAM] = 3.3 μM or none, [Cu^{2+}] = 13.2 μM or none. Images (A) and (C) showed aggregation.

A) *E. coli* (2.3 × 10^7 CFU·mL^{-1}) + Cu-dpa-B-PAMAM

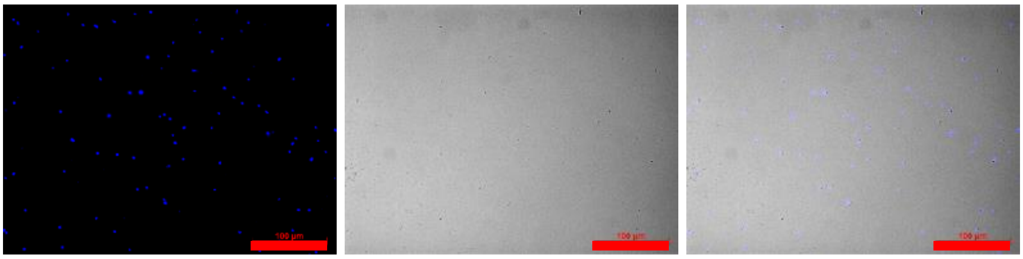

B) *E. coli* (2.3 × 10^7 CFU·mL^{-1})

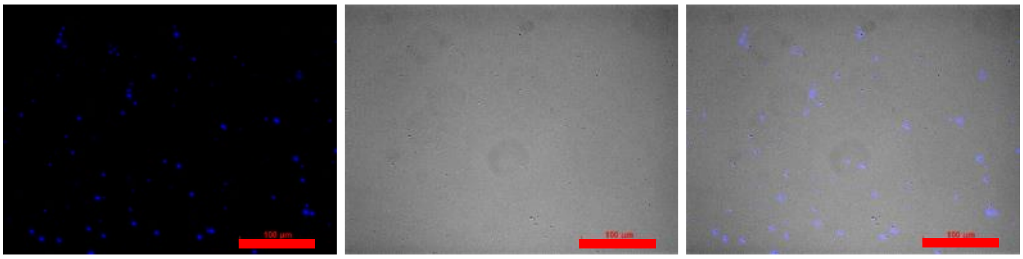

C) *E. coli* (2.3 × 10^5 CFU·mL^{-1}) + Cu-dpa-B-PAMAM

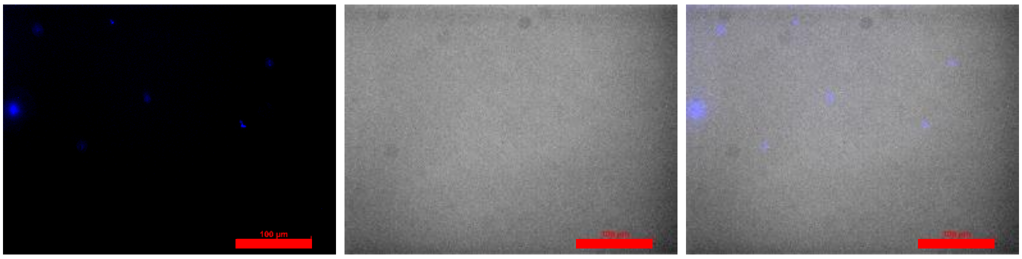

D) *E. coli* (2.3 × 10^5 CFU·mL^{-1})

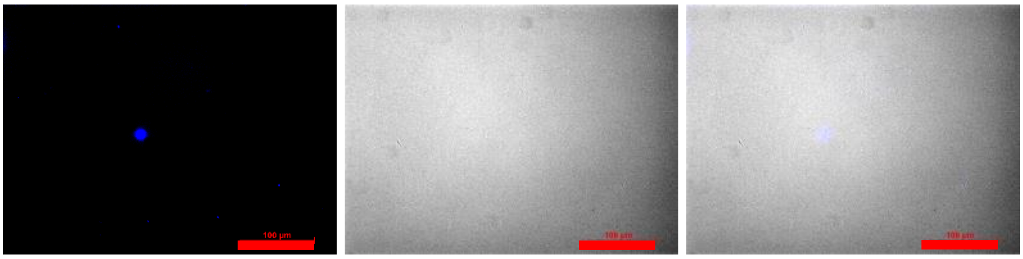

Figure S5. Fluorescent microscopy images in low *E. coli* concentrations at pH 7.4 adjusted with HEPES buffer. [HEPES] = 5.0 mM, the figures (from left to right) demonstrated DAPI, DIC, and merged images. Scale bar was set to 100 μm. A,B) [*E. coli*] = 2.3 × 10^7 CFU·mL^{-1}, [Cu-dpa-B-PAMAM] = 3.3 μM or none, [Cu^{2+}] = 13.2 μM or none. C,D) [*E. coli*] = 2.3 × 10^5 CFU·mL^{-1}, [Cu-dpa-B-PAMAM] = 3.3 μM or none, [Cu^{2+}] = 13.2 μM or none. All images (A-D) did not show aggregation.

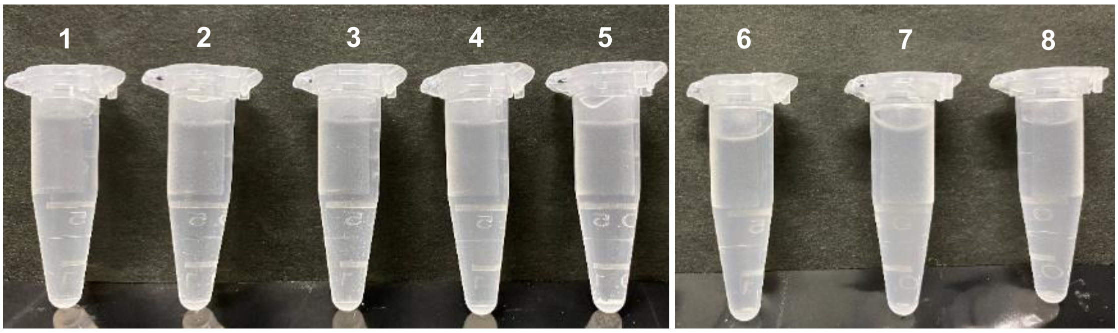

Figure S6. The images of aggregation between Cu-dpa-B-PAMAM and various bacteria. [Cu-dpa-B-PAMAM] = 3.3 μM, [Cu^{2+}] = 13.2 μM, bacteria concentration was set at OD_{600} = 0.3, [HEPES] = 5.0 mM, pH was adjusted at 7.4 with HEPES buffer. 1: *S. aureus* ATCC25923, 2: *S. aureus* ATCC29213, 3: *S. pseudintermedius*, 4: *S. epidermidis*, 5: *E. faecalis*, 6: *E. coli* ATCC25922, 7: *P. aeruginosa*, 8: *S. enteritidis*. The results of turbidity measurement are demonstrated in Figure 6.

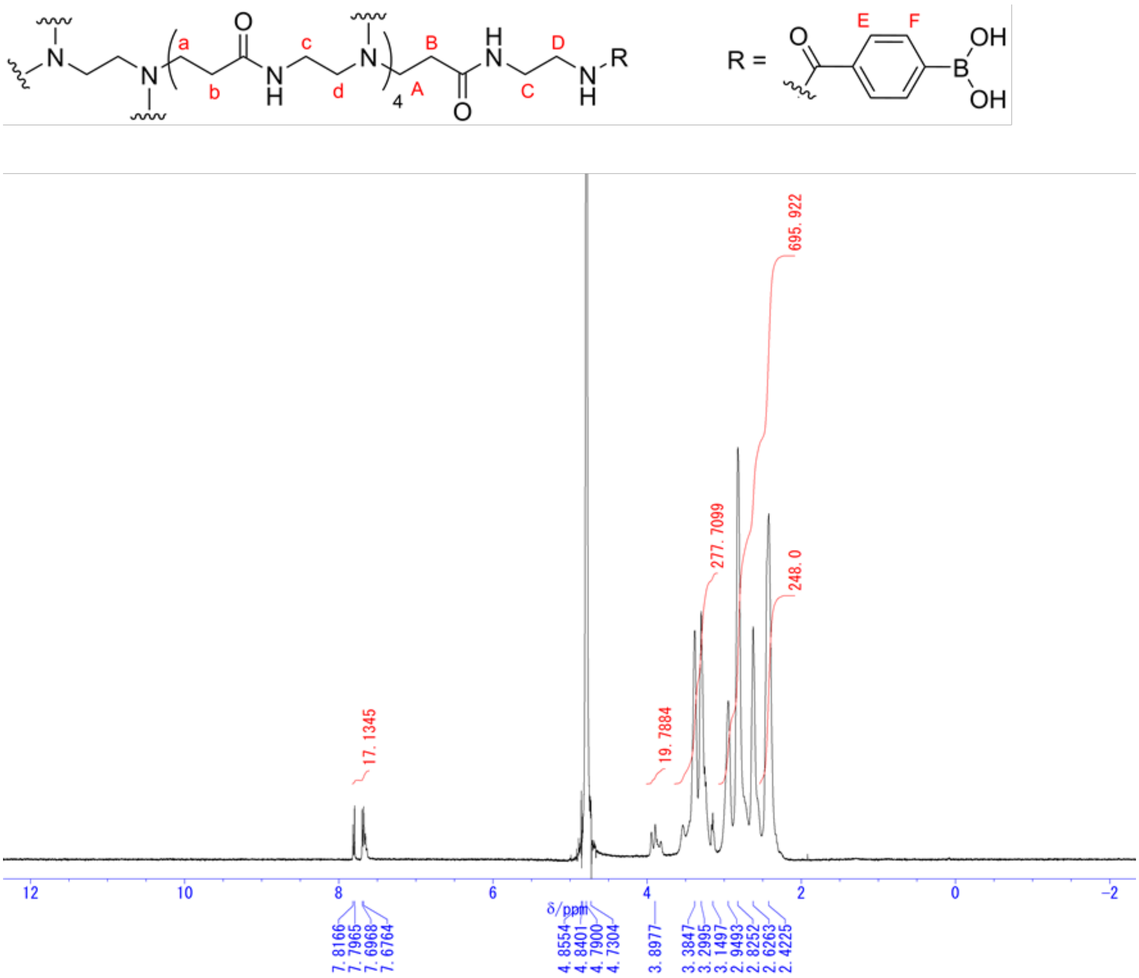

^{1}H NMR (500 MHz, D_2O) δ(ppm): 7.81 (br, 4H, $H_{E,F}$), 7.68 (br, 4H, $H_{E,F}$), 3.90 (br, 744H, $H_{A,a,C,c,D,d}$), 3.38 (br, 744H, $H_{A,a,C,c,D,d}$), 3.30 (br, 744H, $H_{A,a,C,c,D,d}$), 3.15 (br, 744H, $H_{A,a,C,c,D,d}$), 2.95 (br, 744H, $H_{A,a,C,c,D,d}$), 2.83 (br, 744H, $H_{A,a,C,c,D,d}$), 2.63 (br, 744H, $H_{A,a,C,c,D,d}$), 2.42 (br, 248H, $H_{B,b}$),

(E,F):(B+b) = (4M):248 (M: The number of modified phenyl group)

17.1345:248 = (4M):248

M = 4.3

Figure S7. ^{1}H NMR spectrum of B4-PAMAM.

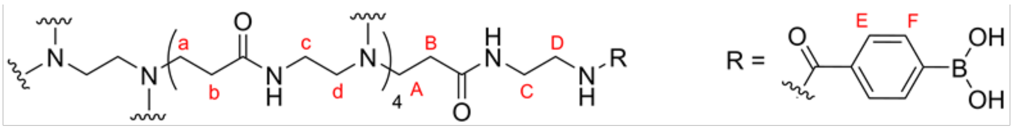

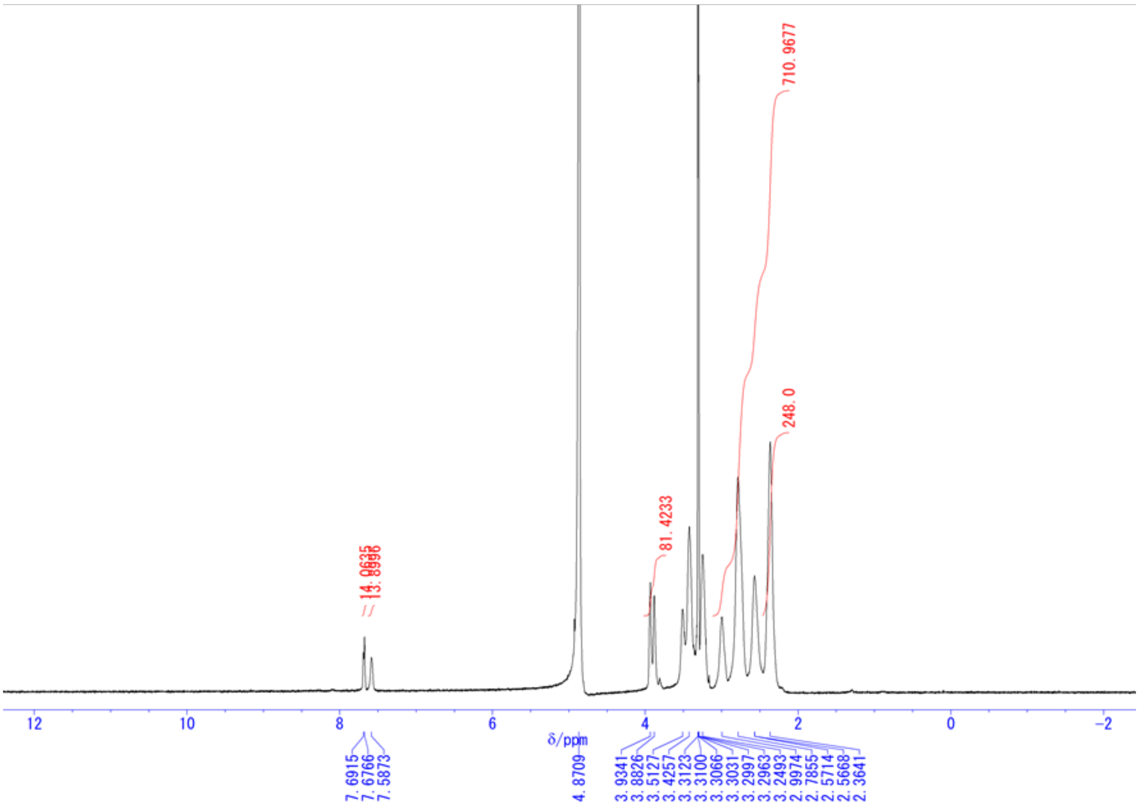

^{1}H NMR (500 MHz, CD_3OD) δ(ppm): 7.68 (br, 4H, $H_{E,F}$), 7.59 (br, 4H, $H_{E,F}$), 3.93 (br, 744H, $H_{A,a,C,c,D,d}$), 3.88 (br, 744H, $H_{A,a,C,c,D,d}$), 3.51 (br, 744H, $H_{A,a,C,c,D,d}$), 3.43 (br, 744H, $H_{A,a,C,c,D,d}$), 3.30 (br, 744H, $H_{A,a,C,c,D,d}$), 3.00 (br, 744H, $H_{A,a,C,c,D,d}$), 2.79 (br, 744H, $H_{A,a,C,c,D,d}$), 2.57 (br, 744H, $H_{A,a,C,c,D,d}$), 2.36 (br, 248H, $H_{B,b}$)

(E,F):(B+b) = (4M):248 (M: The number of modified phenyl group)

27.9631:248 = (4M):248

M = 7.0

Figure S8. ^{1}H NMR spectrum of B7-PAMAM.

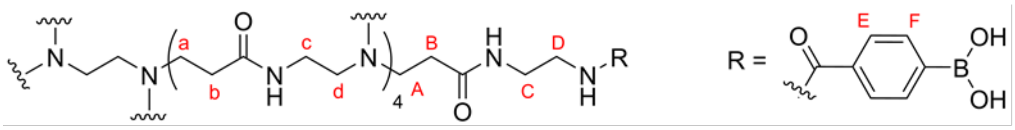

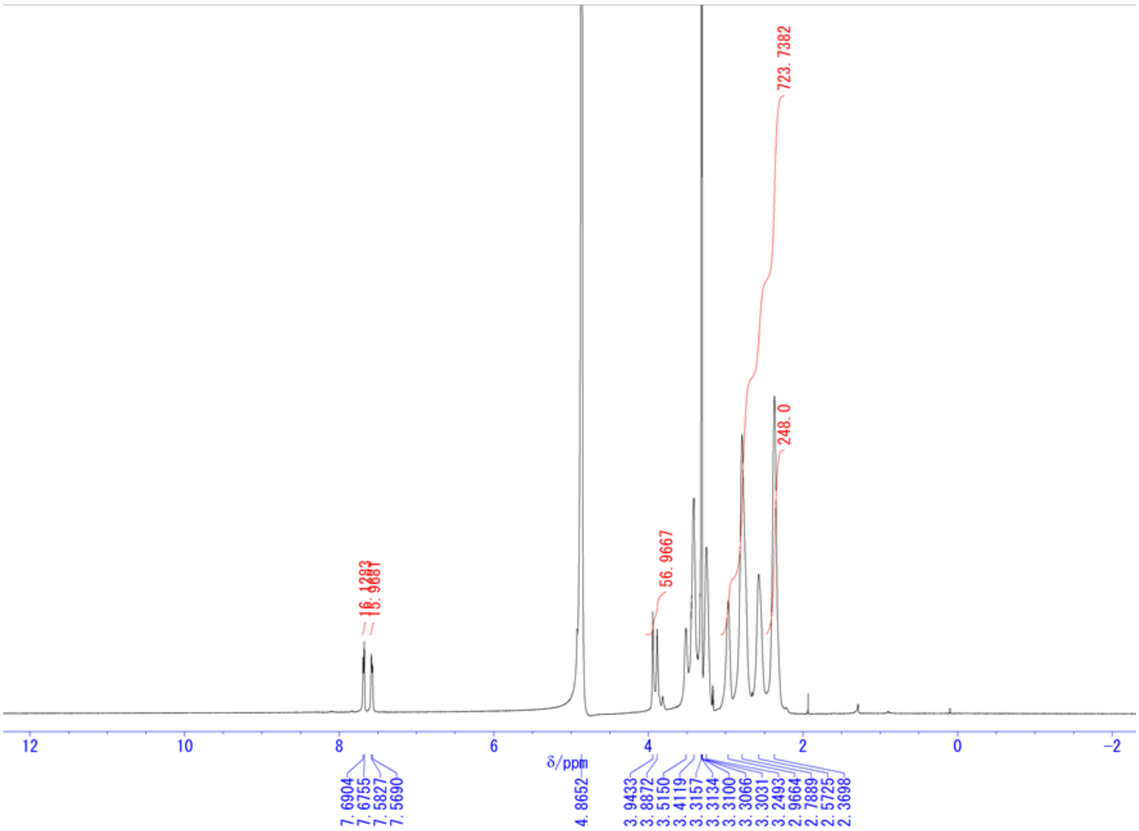

^{1}H NMR (500 MHz, CD_3OD) δ(ppm): 7.68 (br, 4H, $H_{E,F}$), 7.57 (br, 4H, $H_{E,F}$), 3.94 (br, 744H, $H_{A,a,C,c,D,d}$), 3.89 (br, 744H, $H_{A,a,C,c,D,d}$), 3.52 (br, 744H, $H_{A,a,C,c,D,d}$), 3.42 (br, 744H, $H_{A,a,C,c,D,d}$), 3.31 (br, 744H, $H_{A,a,C,c,D,d}$), 2.97 (br, 744H, $H_{A,a,C,c,D,d}$), 2.79 (br, 744H, $H_{A,a,C,c,D,d}$), 2.57 (br, 744H, $H_{A,a,C,c,D,d}$), 2.37 (br, 248H, $H_{B,b}$)

(E,F):(B+b) = (4M):248 (M: The number of modified phenyl group)

32.0964:248 = (4M):248

M = 8.0

Figure S9. ^{1}H NMR spectrum of B8-PAMAM.

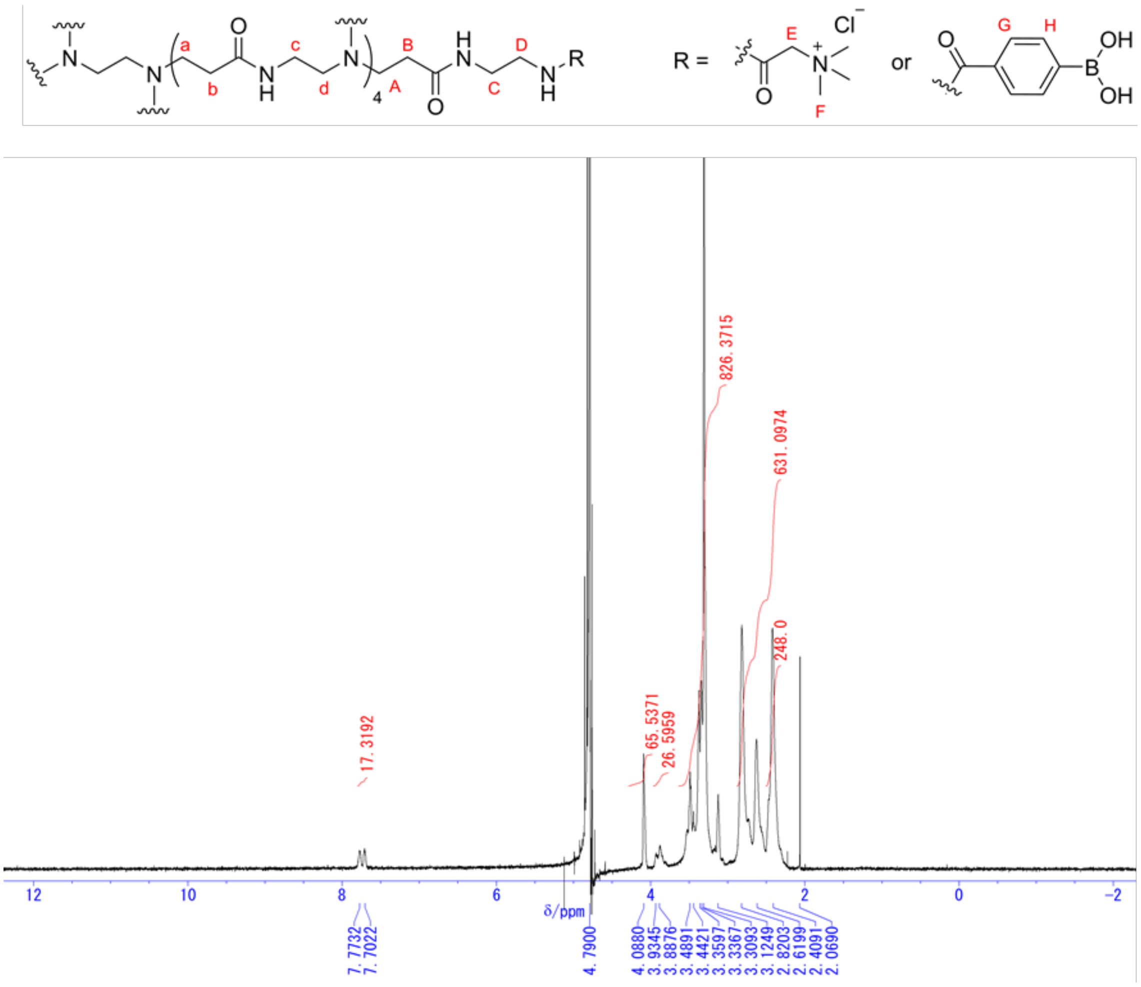

^{1}H NMR (500 MHz, CD_3OD) δ(ppm): 7.77 (br, 4H, $H_{G,H}$), 7.70 (br, 4H, $H_{G,H}$), 4.09 (br, 2H, H_E), 3.89 (br, 744~753H, $H_{A,a,C,c,D,d,F}$), 3.49 (br, 744~753H, $H_{A,a,C,c,D,d,F}$), 3.44 (br, 744~753H, $H_{A,a,C,c,D,d,F}$), 3.36 (br, 744~753H, $H_{A,a,C,c,D,d,F}$), 3.31 (br, 744~753H, $H_{A,a,C,c,D,d,F}$), 3.12 (br, 744~753H, $H_{A,a,C,c,D,d,F}$), 2.82 (br, 744~753H, $H_{A,a,C,c,D,d,F}$), 2.62 (br, 744~753H, $H_{A,a,C,c,D,d,F}$), 2.41 (br, 248H, $H_{B,b}$)

(E):(B+b) = (2M):248 (M: The number of modified betaine group)

65.5371:248 = (2M):248

M = 32.8

Figure S10. ^{1}H NMR spectrum of Bt33-B4-PAMAM.

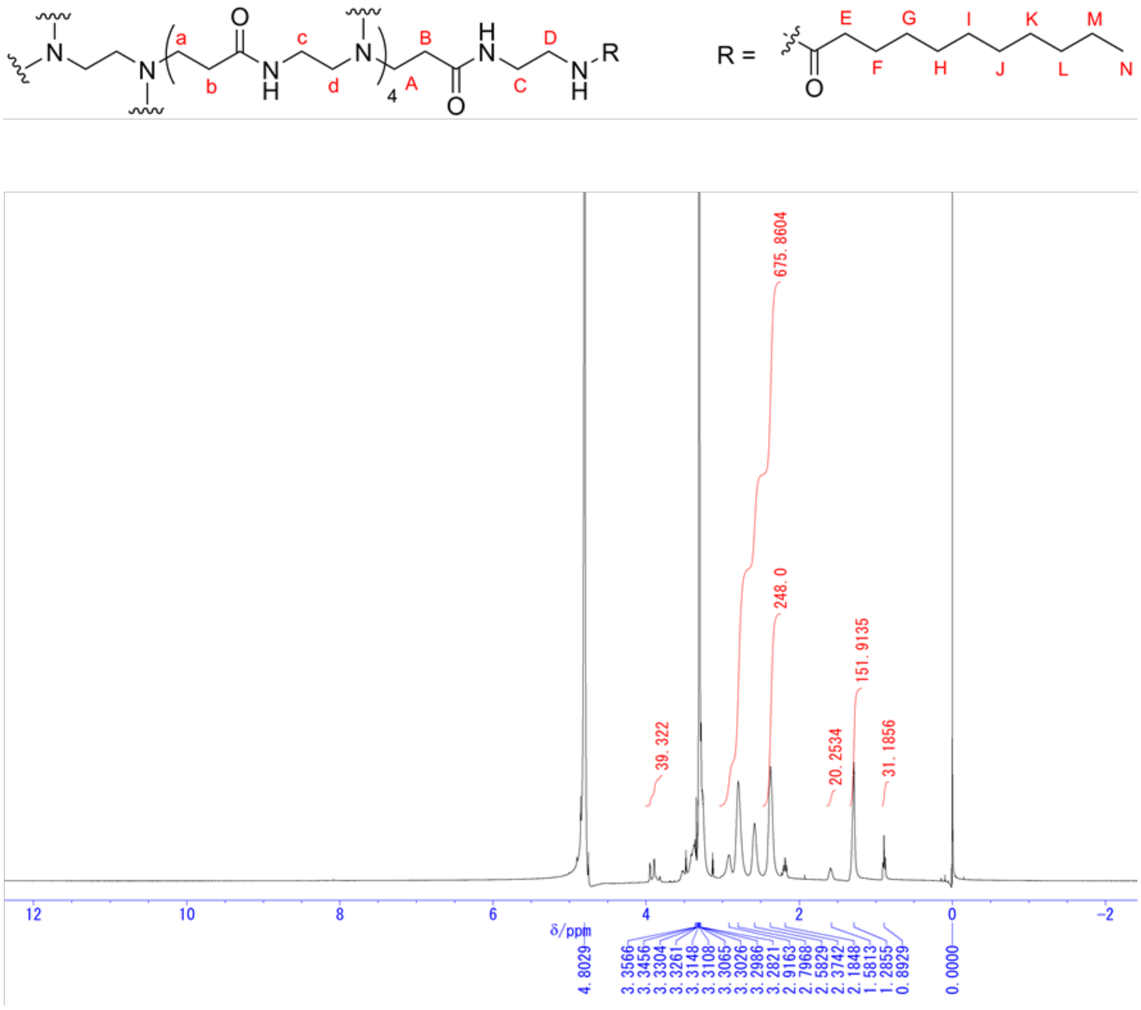

^{1}H NMR (400 MHz, CD_3OD) δ(ppm): 3.93 (br, 744H, $H_{A,a,C,c,D,d}$), 3.33 (br, 744H, $H_{A,a,C,c,D,d}$), 2.92 (br, 744H, $H_{A,a,C,c,D,d}$), 2.80 (br, 744H, $H_{A,a,C,c,D,d}$), 2.58 (br, 744H, $H_{A,a,C,c,D,d}$), 2.37 (br, 744H, $H_{A,a,C,c,D,d}$), 2.18 (br, 744H, $H_{A,a,C,c,D,d}$), 1.58 (br, 2H, H_E), 1.29 (br, 16H, $H_{F\sim M}$), 0.89 (br, 3H, H_N)

(E):(B+b) = (2M):248 (M: The number of modified alkyl group)

20.2354:248 = (2M):248

M = 10.1

Figure S11. ^{1}H NMR spectrum of C10-PAMAM.

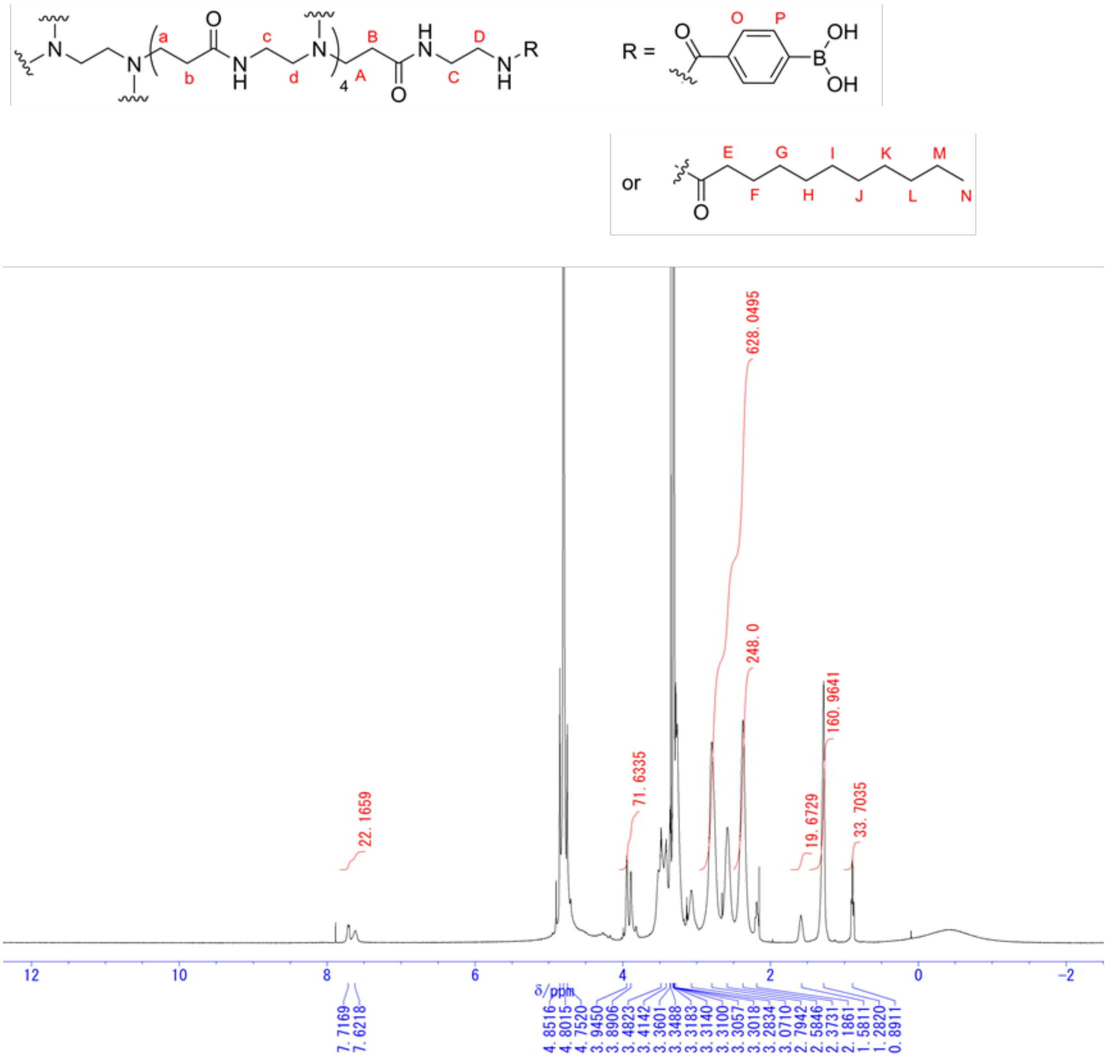

^{1}H NMR (400 MHz, CD_3OD) δ(ppm): 7.72 (br, 4H, $H_{O,P}$), 7.62 (br, 4H, $H_{O,P}$), 3.95 (br, 744H, $H_{A,a,C,c,D,d}$), 3.89 (br, 744H, $H_{A,a,C,c,D,d}$), 3.36 (br, 744H, $H_{A,a,C,c,D,d}$), 3.07 (br, 744H, $H_{A,a,C,c,D,d}$), 2.79 (br, 744H, $H_{A,a,C,c,D,d}$), 2.58 (br, 744H, $H_{A,a,C,c,D,d}$), 2.37 (br, 744H, $H_{A,a,C,c,D,d}$), 2.19 (br, 744H, $H_{A,a,C,c,D,d}$), 1.58 (br, 2H, H_E), 1.28 (br, 16H, $H_{F\sim M}$), 0.89 (br, 3H, H_N)

(O+P):(B+b) = (4M):248 (M: The number of modified phenyl group)
22.1659:248 = (4M):248
M = 5.5

Figure S12. ^{1}H NMR spectrum of C10-B6-PAMAM.

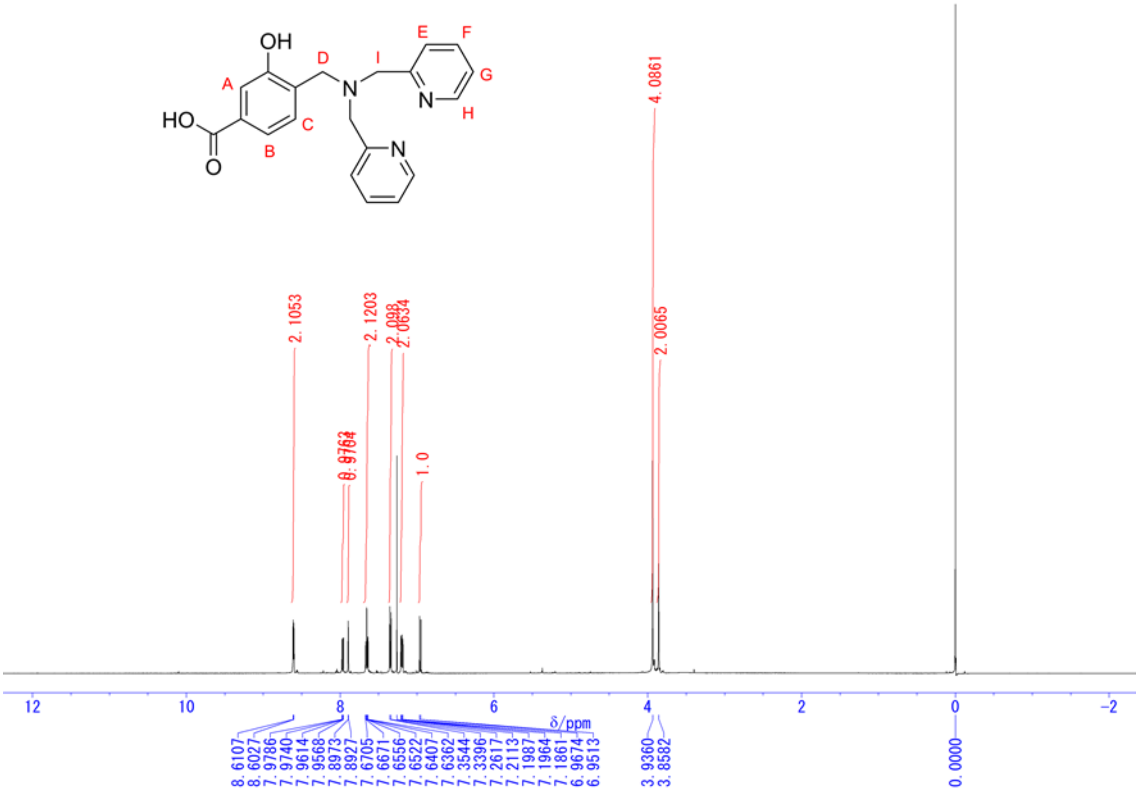

^{1}H NMR (500 MHz, $CDCl_3$) δ(ppm): 8.61 (d, *J* = 4.0 Hz, 2H, H_H), 7.96 (dd, *J* = 2.3, 4.3 Hz, 1H, H_B), 7.89 (d, *J* = 2.3 Hz, 1H, H_A), 7.65 (dt, *J* = 1.7, 7.5 Hz, 2H, H_F), 7.35 (d, *J* = 7.4 Hz, 2H, H_E), 7.20 (dd, *J* = 6.3, 1.2 Hz, 2H, H_G), 6.96 (d, *J* = 8.1 Hz, 1H, H_C), 3.94 (s, 4H, H_I), 3.86 (s, 2H, H_D)

Figure S13. ^{1}H NMR spectrum of dpa.

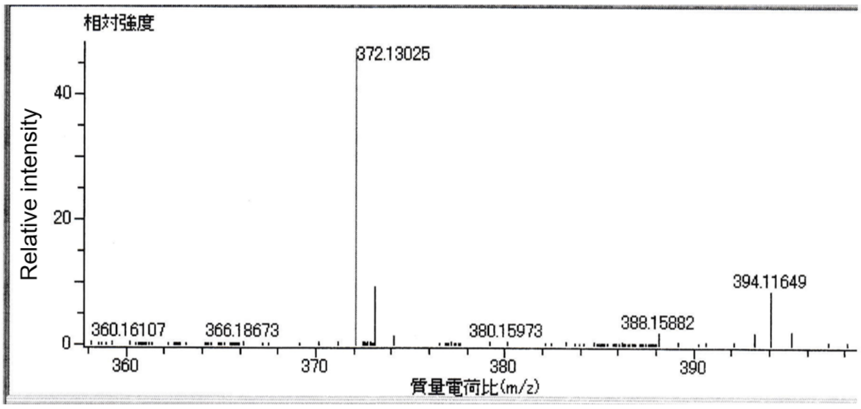

Obsd. *m*/*z*<br>質量	Intensity<br>強度	Calcd. *m*/*z*<br>計算質量	Mass Error<br>質量差 mmu	Mass Accuracy<br>質量差 ppm	Estimated Formula<br>推定組成式	Unsaturation Number<br>不飽和数
372.13025	90921.00	372.13241	-2.16	-5.82	12C20 1H19 14N3 23Na 16O3	12.5
		372.12626	3.98	10.70	12C26 1H16 14N2 16O	20.0
		372.13482	-4.57	-12.28	12C22 1H18 14N3 16O3	15.5

Figure S14. ESI-HRMS spectrum of dpa.

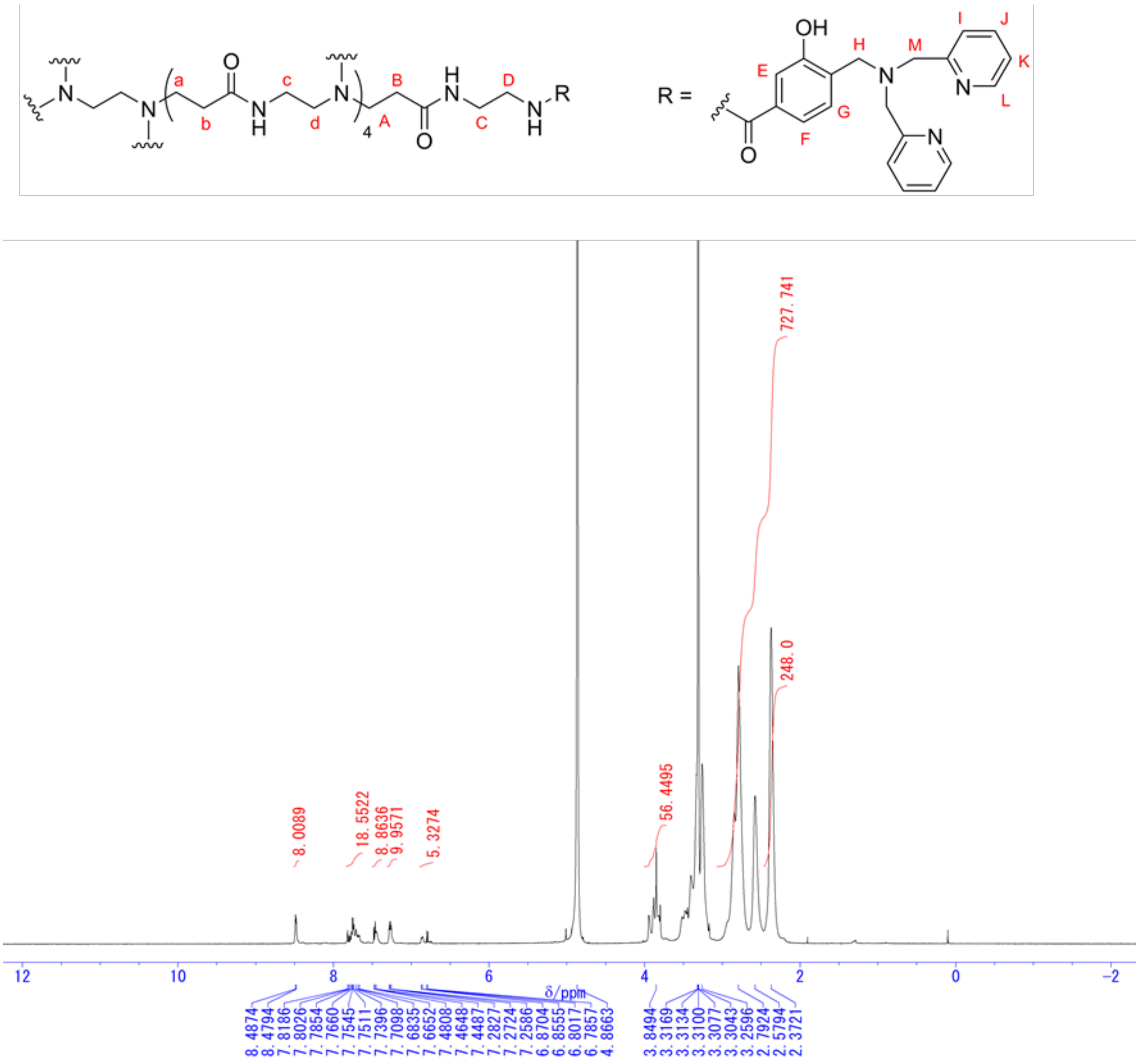

^{1}H NMR (500 MHz, CD_3OD) δ(ppm): 8.48 (br, 2H, H_L), 7.77 (br, 4H, $H_{E,F,J}$), 7.46 (br, 2H, H_I), 7.27 (br, 2H, H_K), 6.86 (br, 1H, H_G), 3.84 (br, 744H, $H_{A,a,C,c,D,d,H,M}$), 3.25 (br, 744H, $H_{A,a,C,c,D,d}$), 2.79 (br, 744H, $H_{A,a,C,c,D,d}$), 2.58 (br, 744H, $H_{A,a,C,c,D,d}$), 2.37 (br, 248H, $H_{B,b}$)

(L):(B+b) = (2M):248 (M: The number of modified dpa group)

8.0089:248 = (2M):248

M = 4.0

Figure S15. ^{1}H NMR spectrum of dpa4-PAMAM.

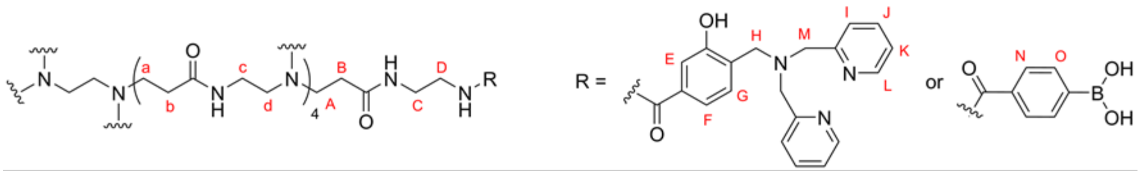

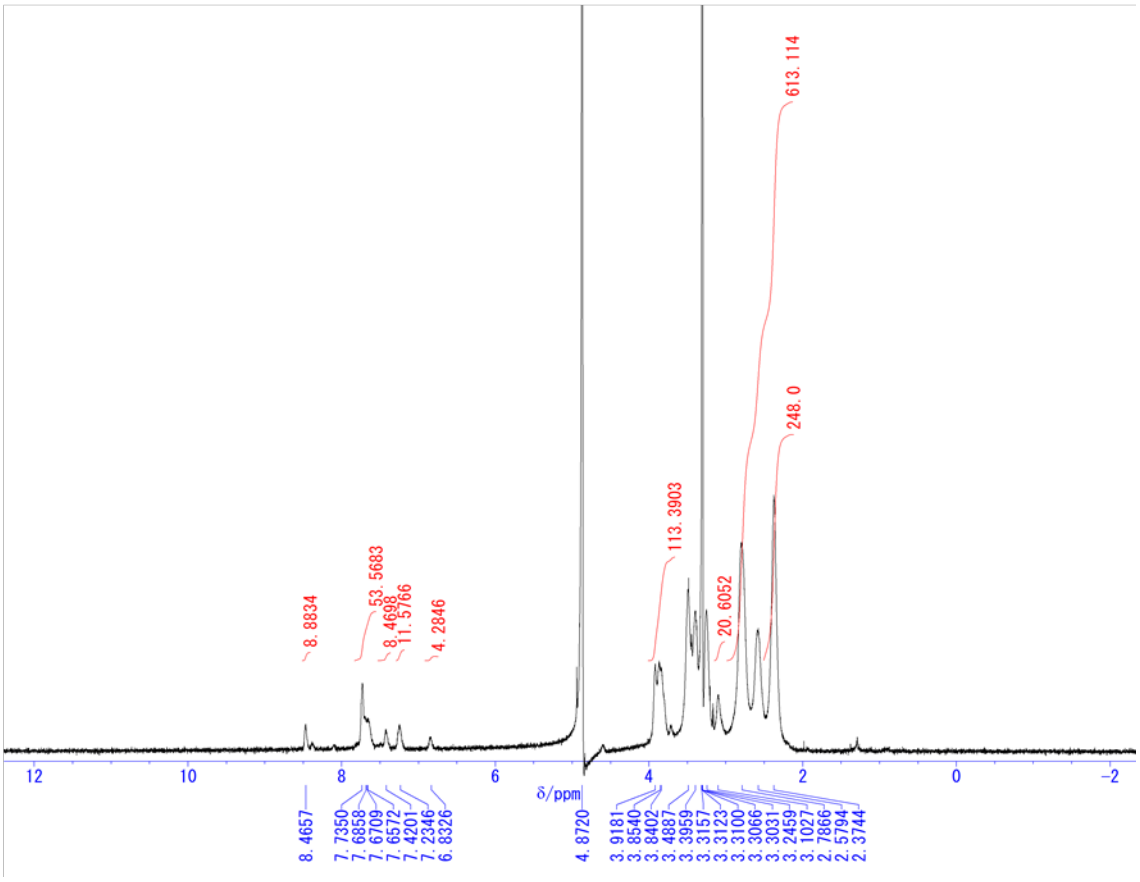

^{1}H NMR (500 MHz, CD_3OD) δ(ppm): 8.47 (br, 2H, H_L), 7.73 (br, 8H, $H_{E,F,J,N,O}$), 7.42 (br, 2H, H_I), 7.23 (br, 2H, H_K), 6.83 (br, 1H, H_G), 3.85 (br, 744H, $H_{A,a,C,c,D,d,H,M}$), 3.25 (br, 744H, $H_{A,a,C,c,D,d}$), 3.10 (br, 744H, $H_{A,a,C,c,D,d}$), 2.79 (br, 744H, $H_{A,a,C,c,D,d}$), 2.58 (br, 744H, $H_{A,a,C,c,D,d}$), 2.37 (br, 248H, $H_{B,b}$)

(L):(B+b) = (2M):248 (M: The number of modified dpa group)

8.8834:248 = (2M):248

M = 4.4

Figure S16. ^{1}H NMR spectrum of dpa4-B7-PAMAM.
